# Metaplastic Breast Carcinoma: Clinicopathological Characteristics, Treatment Patterns, and Outcomes

**DOI:** 10.7759/cureus.106353

**Published:** 2026-04-03

**Authors:** Ulfat Ara, Umeek Jeelani, Basharat Ara Wani, Shahida Nasreen, Lekshmi R Shenoi, Shaheen Nazir Lone, Kaneez Fatima, Shadab Maqsood, Faisal Rashid Guru, Hussain Mir, Asifa Andleeb

**Affiliations:** 1 Medical Oncology, Sher-i-Kashmir Institute of Medical Sciences, Srinagar, IND; 2 Health and Medical Education, Gousiya Hospital, Srinagar, IND; 3 Biochemistry, Government Medical College (GMC), Srinagar, IND; 4 Radiation Oncology, Sher-i-Kashmir Institute of Medical Sciences, Srinagar, IND; 5 Medical Oncology, Yenepoya Medical College, Mangalore, IND; 6 Gastroenterology, Sher-i-Kashmir Institute of Medical Sciences, Srinagar, IND; 7 Radiology, Sher-i-Kashmir Institute of Medical Sciences, Srinagar, IND

**Keywords:** breast-conserving surgery, mastectomy, metaplastic breast carcinoma, neoadjuvant chemotherapy, pathological complete response

## Abstract

Background: Metaplastic breast carcinoma (MBC) is a rare and aggressive subtype of breast cancer, accounting for less than 1% of all cases. MBC is characterized by a metaplastic transformation of glandular epithelium into squamous and/or mesenchymal elements. Most tumors are triple-negative, lacking expression of estrogen receptor (ER), progesterone receptor (PR), and human epidermal growth factor receptor 2 (HER2), which limits targeted therapeutic options. MBC also exhibits marked histopathological heterogeneity and high chemoresistance.

Methods: We conducted a retrospective study of 15 patients with histopathologically confirmed MBC treated at a tertiary care center in India. All cases demonstrated a triple-negative immunophenotype (ER < 1%, PR < 1%, HER2-negative), with documented metaplastic differentiation. Detailed data on demographics, clinicopathological features, treatment, and outcomes were collected and analyzed.

Results: The median age at diagnosis was 52 years. All patients presented with advanced disease: stage II in 60% (n = 9) and stage III in 40% (n = 6). Metaplastic differentiation included spindle cell features in 40% (n = 6), squamous features in 33.3% (n = 5), and mixed patterns in 26.7% (n = 4). Tumors were more commonly located in the right breast (73.3%, n = 11). Surgical management included mastectomy in 60% (n = 9) and breast-conserving surgery in 26.7% (n = 4). Neoadjuvant chemotherapy (NAC) was administered to six patients (40%), achieving a pathological complete response (pCR) in two of these (33.3%). Adjuvant chemotherapy was given to seven patients (46.7%), and 11 patients (73.3%) received radiotherapy. At the last follow-up, six (40%) were alive and disease-free, two (13.3%) were alive with disease, three (20%) had died, two (13.3%) were lost to follow-up, and two (13.3%) had incomplete assessment.

Conclusions: MBC in this cohort presented with aggressive histopathological features and predominantly advanced-stage disease. These findings highlight the need for improved breast cancer awareness, early detection, and individualized treatment strategies, particularly in resource-limited settings.

## Introduction

Breast cancer is the most prevalent malignancy among women worldwide and the second leading cause of cancer-related mortality [1,2]. The burden is particularly high in developing countries, where incidence and mortality rates continue to rise due to limited screening programs, delayed diagnosis, and restricted access to therapeutics [3]. Metaplastic breast carcinoma (MBC) is a rare and heterogeneous group of malignancies, characterized by differentiation of neoplastic epithelium into squamous cells and/or mesenchymal elements, such as spindle cell morphology, chondroid differentiation, or osseous differentiation, among other matrix-producing phenotypes [4,5]. MBC accounts for less than 1% of invasive breast cancers, making it an exceptionally rare malignancy that poses multiple diagnostic and therapeutic challenges [6,7].

Most MBCs are immunohistochemically triple-negative, lacking expression of estrogen receptor (ER), progesterone receptor (PR), and human epidermal growth factor receptor 2 (HER2) [8,9]. Despite this, MBC demonstrates distinct biological behavior, including increased chemoresistance, larger tumor size at presentation, paradoxically lower rates of lymph node metastasis despite aggressive local behavior, and characteristic patterns of distant metastasis [10,11]. Histological subtypes, as defined by the World Health Organization (WHO), include low-grade adenosquamous carcinoma, fibromatosis-like metaplastic carcinoma, spindle cell carcinoma, squamous cell carcinoma, and metaplastic carcinoma with mesenchymal differentiation [4,12]. Molecular alterations are common in MBC. Phosphoinositide 3-kinase/protein kinase B/mammalian target of rapamycin (PI3K/AKT/mTOR) pathway alterations occur in approximately 57% of cases, with PIK3CA mutations present in 29%, varying by histological subtype [9]. These mutations activate pro-survival and proliferative signaling pathways, contributing to therapy resistance. Additionally, MBC frequently overexpresses epidermal growth factor receptor (EGFR), particularly in spindle cell and squamous subtypes. Epithelial-mesenchymal transition (EMT), involving loss of epithelial polarity and acquisition of mesenchymal properties, is a molecular hallmark of metaplastic differentiation [9,13].

Unlike hormone receptor-positive or HER2-positive breast cancers, which are amenable to targeted therapies, MBC lacks targetable receptors and relies primarily on cytotoxic chemotherapy [14]. However, MBC is often resistant to anthracycline-taxane regimens, with reported pathological complete response (pCR) rates of 0%-10% following neoadjuvant chemotherapy (NAC) [15,16]. Chemoresistance is attributed to EMT features, PI3K pathway activation, and enhanced DNA repair mechanisms [17]. Prognosis is generally poor, with five-year overall survival (OS) rates ranging from 40% to 65% [6,18,19]. The relatively low incidence of lymph node involvement, despite aggressive disease, suggests a predominance of hematogenous dissemination, potentially linked to EMT-mediated alterations in metastatic behavior [20]. Breast cancer remains under-investigated in developing nations, particularly in regions with limited healthcare infrastructure and cancer screening programs [21,22].

This retrospective study aimed to systematically describe the demographic characteristics, clinicopathological features, histological subtype distribution, treatment modalities, and clinical outcomes of patients with MBC treated at a tertiary care center in India. The findings provide real-world insights into the challenges of managing this rare malignancy in resource-limited settings and contribute to the sparse literature on MBC in underserved populations in low- and middle-income countries (LMICs).

## Materials and methods

Study design and setting

This retrospective study was conducted at the Sher-i-Kashmir Institute of Medical Sciences (SKIMS), Soura, Srinagar, a tertiary care oncology center in India. Patients treated between January 2015 and December 2022 were included. The study systematically evaluated the clinicopathological characteristics, treatment patterns, and clinical outcomes of patients with histologically and immunohistochemically confirmed MBC managed at this institution.

Patient population

This retrospective study included 15 patients with histologically confirmed MBC. The majority (n = 12, 80%) resided in rural areas, while three patients (20%) were from urban areas at the time of diagnosis. This distribution reflects the natural catchment population of the center. The predominance of rural patients allowed the assessment of MBC presentation patterns and outcomes in a population with limited access to specialized cancer care, early detection programs, and advanced diagnostic resources.

Inclusion Criteria

Patients were eligible if they met all of the following criteria: (1) histologically confirmed MBC according to WHO classification, demonstrating metaplastic components on pathological examination [4]; (2) triple-negative status confirmed by immunohistochemistry (IHC) (ER < 1% nuclear staining, PR < 1% nuclear staining, HER2-negative defined as IHC 0/1+ or IHC 2+ with negative fluorescence in situ hybridization); (3) age ≥ 18 years at diagnosis; and (4) treatment at the study institution with sufficient medical record documentation.

Exclusion Criteria

Patients were excluded if they lacked complete immunohistochemical data or had insufficient medical record documentation for analysis.

Medical records were systematically reviewed, and comprehensive data were extracted, including demographics (age at diagnosis, geographic location); clinical presentation (duration of symptoms, tumor laterality); pathological characteristics (tumor size, lymph node involvement, tumor stage, histological grade (according to the modified Bloom-Richardson system), metaplastic subtype); presence or absence of lymphovascular invasion (LVI); Ki-67 proliferation index; treatment modalities (type of surgical procedure, NAC regimens and cycles, adjuvant chemotherapy regimens and cycles, and radiation therapy); pathological response (pCR, defined as ypT0/is ypN0); and clinical outcomes (recurrence status, recurrence sites, vital status at last follow-up, and date of last follow-up or death).

Clinical staging and pathological assessment

Clinical staging was performed according to the tumor, lymph node, metastasis (TNM) staging system of the American Joint Committee on Cancer (AJCC) Cancer Staging Manual, Eighth Edition [23], using physical examination and standard imaging, including mammography and ultrasonography. Computed tomography (CT) was obtained when clinically indicated to assess for distant metastasis.

MBC was diagnosed by experienced pathologists based on hematoxylin and eosin (H&E)-stained sections, demonstrating invasive carcinoma with squamous and/or mesenchymal metaplastic differentiation, in accordance with the WHO classification. IHC was performed on formalin-fixed, paraffin-embedded tissue sections according to institutional protocols. Triple-negative status was defined as ER and PR < 1% nuclear staining and HER2 negativity. The Ki-67 proliferation index was classified as low (<20%), intermediate (20%-30%), or high (>30%). LVI was assessed on H&E-stained sections.

Treatment protocols

Treatment recommendations were determined by a multidisciplinary tumor board, including surgical, medical, and radiation oncologists, as well as pathologists. Patients with clinical stage IIB-IIIC disease or those requiring tumor downstaging to facilitate breast-conserving surgery received NAC. NAC regimens were anthracycline-based (doxorubicin-cyclophosphamide), followed by paclitaxel or docetaxel, with platinum (carboplatin) added in selected cases based on tumor biology and multidisciplinary discussion. Surgical therapy included modified radical mastectomy, simple mastectomy, or breast-conserving surgery (lumpectomy or wide local excision), with axillary lymph node evaluation, determined by tumor characteristics, response to NAC, patient preference, and the feasibility of achieving negative margins. Patients undergoing upfront surgery without NAC or those with residual disease after NAC received adjuvant chemotherapy, typically an anthracycline-taxane combination or fluorouracil-epirubicin-cyclophosphamide regimen, administered over 4-6 cycles. Radiation therapy was delivered according to standard guidelines, including post-mastectomy radiation for high-risk features (T3/T4 tumors, ≥4 positive lymph nodes, or positive margins) and adjuvant whole-breast radiation following breast-conserving surgery.

Outcome measures

Pathological response was defined as the absence of invasive carcinoma in both the breast and axillary lymph nodes (ypT0/is ypN0) on the final pathology following NAC. Disease-free survival (DFS) was calculated from the date of definitive surgery to the date of the first documented recurrence (local, regional, or distant) or the last follow-up in patients without recurrence. OS was calculated from the date of diagnosis to the date of death or last follow-up for surviving patients. Vital status at last follow-up was classified as alive without disease, alive with disease, deceased, or lost to follow-up.

Statistical analysis

Patient demographics, clinical characteristics, treatment details, and outcomes were summarized using descriptive statistics. Categorical variables were reported as frequencies and percentages, while non-normally distributed continuous variables were presented as medians with interquartile ranges (IQR). Due to the small sample size, a formal Kaplan-Meier survival analysis was not performed. Survival data, including DFS and OS, were reported descriptively as medians with IQR and by categorical vital status at the final follow-up. All statistical analyses were conducted using IBM SPSS Statistics for Windows, Version 25.0 (Released 2017; IBM Corp., Armonk, NY, USA).

Ethical considerations

This study was conducted in accordance with the Declaration of Helsinki and approved by the Institutional Ethics Committee of Sher-i-Kashmir Institute of Medical Sciences (IEC/SKIMS Protocol # 53/2025). The need for informed consent was waived owing to the retrospective nature of the study and the use of anonymized data.

## Results

Patient demographics and clinical characteristics

**Figure 1:**
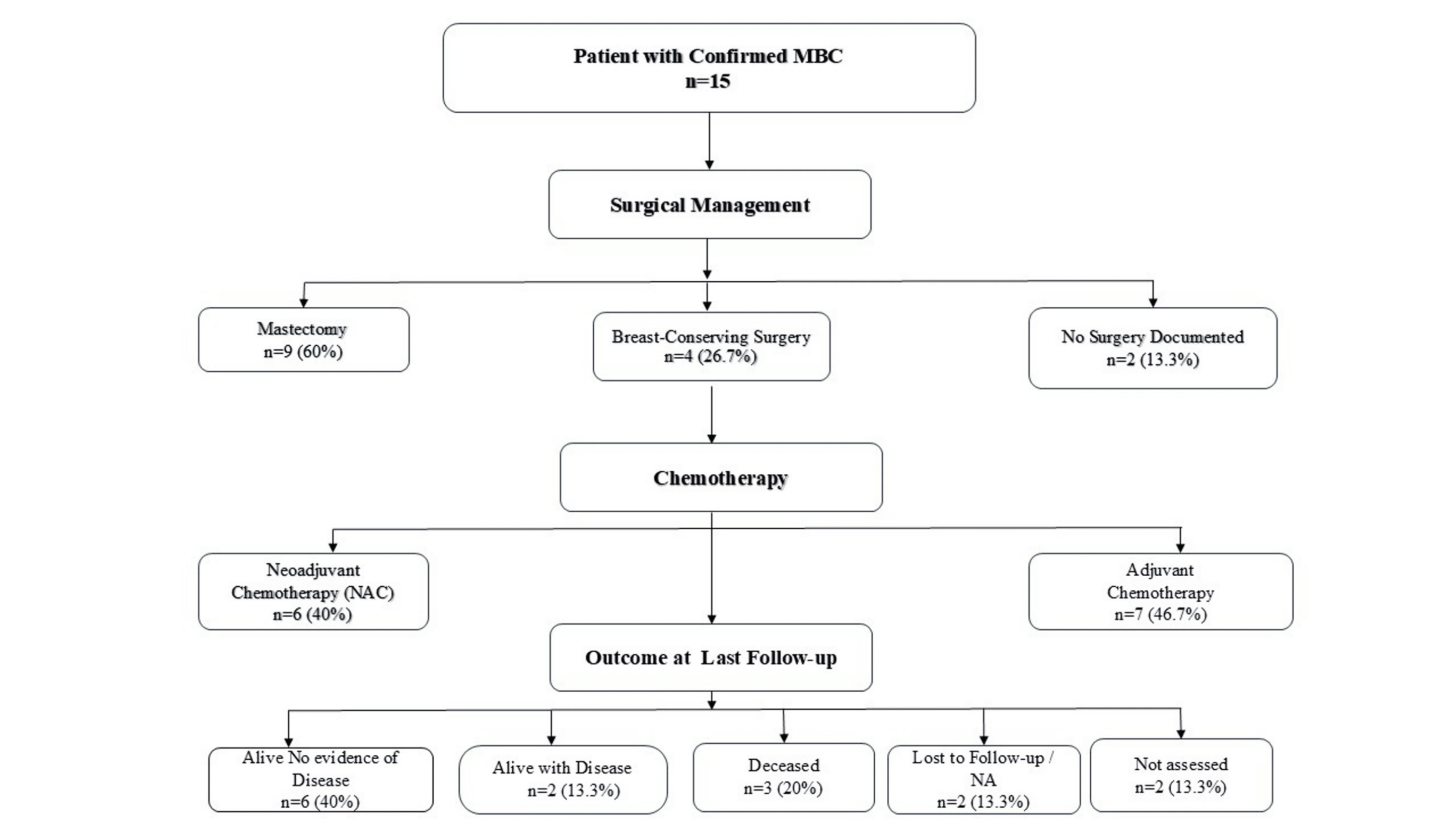
Patient flow diagram

This study included 15 patients with histopathologically confirmed MBC. The demographic characteristics, clinical features, and stage distribution at diagnosis are summarized in Table 1. The patient flow diagram for the study is presented in Figure 1.

**Table 1:** Demographics, including clinical characteristics and stage distribution of MBC patients (N = 15) MBC: metaplastic breast carcinoma.

Characteristics	Value
Age at diagnosis, median (IQR), years	52 (41-63)
Geographic distribution, n (%)	
Rural	12 (80.0)
Urban	3 (20.0)
Tumor laterality, n (%)	
Right	11 (73.3)
Left	4 (26.7)
Symptom duration at presentation, median (IQR), months	4 (2-4.75)
Disease stage distribution at diagnosis	
Substage, n (%)	
Stage II	1 (6.7)
Stage IIA	4 (26.7)
Stage IIB	4 (26.7)
Subtotal - stage II	9 (60.0)
Stage III	1 (6.7)
Stage IIIA	3 (20.0)
Stage IIIB	1 (6.7)
Stage IIIC	1 (6.7)
Subtotal - stage III	6 (40.0)
Total	15 (100.0)

Histopathological classification and metaplastic subtypes

Metaplastic differentiation patterns were systematically classified based on histopathological examination. The most common subtype was spindle cell differentiation, observed in six patients (40%), characterized by proliferation of spindle-shaped cells without distinct mesenchymal lineage differentiation. Squamous differentiation was identified in five patients (33.3%) and showed keratinizing squamous cell morphology. Mixed differentiation patterns, including combinations of multiple metaplastic components and/or chondroid or osseous elements, were observed in four patients (26.7%). All 15 cases demonstrated a triple-negative immunophenotype on IHC.

Tumor characteristics and disease stage

Tumor size was classified according to the primary tumor (T) category of the AJCC TNM staging system [23]. The T2 category was the most common, accounting for 53.3% of cases. Overall, the majority of patients had T2-T4 tumors (86.7%), indicating a predominance of larger and/or locally advanced primary tumors in this cohort. Notably, no patients presented with stage I or stage IV disease, reflecting the tendency toward advanced-stage presentation in populations with limited access to organized screening programs. The TNM stage distribution of patients with MBC is shown in Table 2.

**Table 2:** TNM distribution of MBC patients (N = 15) Note: T and N categories were defined as per the AJCC Cancer Staging Manual, Eighth Edition. Overall, an absence of stage I and stage IV was reported; stage II/III distribution requires full TNM (including M) and stage grouping. TNM: tumor, lymph node, metastasis, MBC: metaplastic breast carcinoma.

Breast cancer presentation	Definition	n (%)
Primary tumor (T)		
T category		
T1	≤2 cm	2 (13.3)
T2	>2-5 cm	8 (53.3)
T3	>5 cm	3 (20.0)
T4	Tumor of any size, with chest wall and/or skin involvement	2 (13.3)
Total		15 (100)
T2-T4 subtotal	Larger and/or locally advanced primary tumors	13 (86.7)
Regional lymph nodes (N)		
N category		
N0	Node-negative	4 (26.7)
N1	1-3 positive nodes	6 (40.0)
N2	4-9 positive nodes	3 (20.0)
N3	≥10 positive nodes and/or specific anatomical locations (per TNM)	2 (13.3)
Total		15 (100)
Node-positive (N1-N3) subtotal	Any regional nodal metastasis	11 (73.3)

Pathological features

The Ki-67 proliferation index was categorized as high (>30%) in 11 patients (73.3%), intermediate (20%-30%) in three patients (20%), and low (<20%) in one patient (6.7%). High Ki-67 expression was predominant, indicating the aggressive proliferative activity characteristic of MBC. LVI was present in eight cases (53.3%), absent in five cases (33.3%), and not reported in two cases (13.3%) due to inadequate tissue sampling. The high frequency of LVI in this cohort further supports the aggressive tumor biology of MBC, which is associated with an increased risk of lymph node metastasis and distant recurrence.

Treatment modalities

Mastectomy was the most common surgical procedure, performed in nine patients, including simple mastectomy (n = 5) and modified radical mastectomy (n = 4). The high mastectomy rate reflected the advanced tumor stage, larger tumor size, and inability to achieve adequate margins with breast-conserving surgery. Breast-conserving surgery was performed in four patients, primarily in cases with smaller tumors or after a favorable response to NAC. Two patients did not undergo surgery owing to patient refusal (n = 1) or medical inoperability (n = 1).

NAC was administered to six patients with locally advanced disease. Regimens consisted of anthracycline-taxane-based therapy (doxorubicin-cyclophosphamide, followed by paclitaxel or docetaxel), with the addition of platinum (carboplatin) in selected cases. pCR, defined as ypT0/is ypN0, was achieved in two patients (33.3% of the NAC group; 13.3% of the total cohort), while residual disease was present in four patients (66.7% of the NAC group). Adjuvant chemotherapy was administered to seven patients who underwent upfront surgery or had residual disease after NAC, using 4-6 cycles of anthracycline-taxane or fluorouracil-epirubicin-cyclophosphamide regimens. Radiation therapy was delivered to 11 patients, including post-mastectomy radiation for high-risk features and adjuvant whole-breast irradiation following breast-conserving surgery. A summary of treatment modalities is presented in Table 3.

**Table 3:** Summary of treatment modalities (N = 15)

Treatment category	
Intervention	n (%)
Surgical management	
Simple mastectomy	5 (33.3)
Modified radical mastectomy	4 (26.7)
Total mastectomy	9 (60.0)
Breast-conserving surgery	4 (26.7)
No surgical intervention	2 (13.3)
Neoadjuvant chemotherapy (NAC)	
Received NAC	6 (40.0)
Pathological complete response	2 (33.3)
Residual disease	4 (66.7)
Did not receive NAC	9 (60.0)
Adjuvant chemotherapy	
Received adjuvant chemotherapy	7 (46.7)
Did not receive adjuvant chemotherapy	8 (53.3)
Radiation therapy	
Received radiotherapy	11 (73.3)
Did not receive radiotherapy	4 (26.7)

Clinical outcomes

At a median follow-up of 18 months (range, 6-36 months) among patients with ongoing follow-up, disease recurrence was observed in five patients. The most frequent metastatic sites were the lung, liver, and brain, with two patients developing metastases at multiple sites. The predominance of distant rather than locoregional recurrence is consistent with the known aggressive metastatic behavior of MBC. Although the median follow-up for the entire cohort was 18 months, the median time to recurrence of 20 months (IQR, 18-24 months) was derived from these five patients, each of whom had individual follow-up durations extending beyond 18 months within the observed range. Patterns of disease recurrence and metastatic sites during follow-up are summarized in Table 4. OS status at the last follow-up is presented in Table 5.

**Table 4:** Disease recurrence and metastatic site patterns during follow-up (N = 15)

Variable/category	n (%)
Disease recurrence	
Any recurrence	5 (33.3)
No recurrence	10 (66.7)
Type of recurrence	
Local recurrence	1 (6.7)
Distant metastasis	4 (26.7)
Sites of distant metastasis	
Lung	2 (13.3)
Liver	2 (13.3)
Brain	1 (6.7)
Multiple metastatic sites	2 (13.3)

**Table 5:** Overall survival status at the last follow-up (N = 15)

Status category	n (%)
Alive with no evidence of disease	6 (40.0)
Alive with disease	2 (13.3)
Deceased	3 (20.0)
Lost to follow-up	2 (13.3)
Not assessed	2 (13.3)
Total	15 (100.0)

## Discussion

The present retrospective study of 15 patients with MBC describes the clinicopathological features, treatment patterns, and outcomes of this rare malignancy in a predominantly rural population with limited resources in India. All patients presented at advanced stages (100% stage II-III) and demonstrated aggressive features, including a high Ki-67 proliferation index, frequent LVI, and diverse metaplastic subtypes, with predominance of spindle cell and squamous differentiation. Treatment responses were heterogeneous, with a pCR observed in 33.3% of patients receiving NAC. The combined rate of loss to follow-up and incomplete assessment was 26.7%, reflecting real-world challenges in continuity of care.

The median age at diagnosis in our study was 52 years, which is comparable to previously published literature, including the institutional study by Hennessy et al., who reported a median age of 48 years, while population-based analyses have demonstrated median ages in the sixth decade of life [24,25]. The distribution of metaplastic subtypes (spindle cell 40%, squamous 33.3%, mixed 26.7%) is consistent with prior reports, in which spindle cell and squamous subtypes are the most frequent [26,27]. All tumors demonstrated high-grade histology, and most had a high Ki-67 proliferation index (>30% in 73.3%), indicating aggressive proliferative activity of MBC and a greater risk of recurrence [20]. LVI was present in 53.3% of cases, somewhat lower than the higher rates reported by Rakha et al. [28], and similarly associated with poor prognosis and increased metastatic risk.

A notable finding was the presentation at an advanced stage (100% stage II-III, no stage I cases), in contrast to Western populations, where 40%-50% of breast cancers present at stage I due to established screening programs [29]. The advanced-stage presentation in our cohort likely reflects multiple factors, including the absence of systematic breast cancer screening in rural India, delayed presentation (median symptom duration of four months), limited availability of diagnostic centers, lack of breast cancer awareness, and the inherently aggressive biology of MBC. The nodal involvement rate in our cohort (73.3%) was higher than that reported in some published MBC series, which describe relatively lower rates of lymph node metastasis [30]; this difference may be related to delayed presentation in a predominantly rural population.

Rural populations in India face several barriers to optimal cancer care, including long distances to tertiary care facilities, limited transportation, financial constraints, inadequate health literacy, cultural barriers to healthcare-seeking behavior, and insufficient healthcare infrastructure.

A significant observation in our study was the pCR rate of 33.3% (two of six patients) among those receiving NAC, which is higher than the 0%-10% rates typically reported for MBC [15,16]. This finding should be interpreted with caution due to the small sample size (n = 6), which introduces considerable statistical variability. Possible explanations include the use of platinum-containing regimens in selected cases, as platinum agents have shown activity in some MBC subsets, with case reports describing near-complete responses to platinum-based neoadjuvant therapy [31,32]. In addition, histological heterogeneity may influence chemosensitivity, as tumors with predominant epithelial differentiation may respond better than those with extensive mesenchymal differentiation. A small sample size may also limit the reliability of population-level response estimates.

Chemoresistance in MBC is well documented and has been linked to EMT, activation of the PI3K/AKT/mTOR pathway, and cancer stem-cell-like characteristics [17,33]. Nevertheless, the achievement of pCR in two patients suggests that a subset of MBC may remain responsive to standard chemotherapy, highlighting the need for predictive biomarkers. Residual disease after NAC was observed in four patients (66.7%), underscoring the need for novel therapeutic strategies.

The management strategies in our cohort were consistent with current standards of care for MBC. The absence of ER, PR, and HER2 expression precludes hormonal or HER2-targeted therapy, making cytotoxic chemotherapy the primary systemic treatment option. Anthracycline and taxane-based regimens formed the basis of neoadjuvant and adjuvant treatment in our cohort, consistent with regimens most commonly employed in previously reported MBC series [15,16]. The high rate of mastectomy (60%) reflects the large tumor size at diagnosis, similar to previous reports by Nelson et al. and Pezzi et al., in which mastectomy was the most common surgical procedure in MBC due to advanced local disease [6,8]. The post-mastectomy radiation rate of 73.3% is consistent with guideline-based indications, as most patients had T3/T4 tumors or multiple positive lymph nodes.

Our cohort, in which 80% of patients were from rural areas, provides insight into the presentation and outcomes of MBC in underserved populations. This distribution reflects the natural catchment area of our institution rather than a predetermined study objective. Rural populations in LMICs face multiple overlapping barriers to optimal cancer care, including limited screening programs, restricted access to specialized oncology services, financial burden related to travel and treatment, geographic barriers to multimodal therapy, limited health literacy, and cultural factors influencing healthcare utilization [21,22].

These systemic barriers likely contributed to the advanced-stage presentation (100% stage II-III) and delayed symptom duration (median four months) observed in our cohort. The relatively high rate of loss to follow-up and incomplete assessment (26.7%) further reflects the difficulty of maintaining long-term cancer care in rural settings, where transportation difficulties, financial limitations, and competing socioeconomic priorities may interfere with follow-up [34]. Despite these challenges, our results suggest that delivery of guideline-based multimodal treatment is feasible, as 40% of patients were disease-free at the last follow-up. However, improvement in outcomes will require strengthening the continuum of care through community-based education, expansion of screening programs, patient navigation services, financial and transportation support, telemedicine-based referral networks, and community health worker involvement to improve adherence to follow-up [35].

This study has several limitations related to the rarity of MBC and the retrospective design. The small sample size (N = 15), although relatively substantial for a tumor accounting for <1% of breast cancers, limits generalizability and precludes robust statistical analysis. Because of the descriptive design and small cohort, no multivariate or inferential statistical testing was performed; such analyses would require larger cohorts to ensure sufficient statistical power.

The single-center retrospective design may introduce selection bias, as patients treated at a tertiary referral center may not represent the broader MBC population. In addition, the relatively short follow-up period (median 18 months) and the proportion of patients lost to follow-up may limit assessment of long-term outcomes and introduce survival bias. Molecular characterization was limited to conventional immunohistochemistry without advanced biomarker analysis, and treatment approaches were not fully standardized, which may confound outcome comparisons and limit interpretation of treatment efficacy.

## Conclusions

This study on MBC highlights the aggressive biology and advanced-stage presentation characteristic of this rare subtype, as reflected by the predominance of stage II-III disease, high-grade histology, and frequent LVI. Although MBC is generally considered chemoresistant, a subset of patients achieved pCR following NAC, suggesting that selected patients may benefit from standard multimodal treatment. These findings should be interpreted with caution and require confirmation in prospective multicenter studies with larger cohorts to establish standardized treatment protocols and to identify predictive biomarkers that may guide personalized therapeutic strategies. Novel therapeutic approaches, including agents targeting the PI3K/AKT/mTOR pathway, immune checkpoint inhibitors, and antibody-drug conjugates, warrant further investigation in patients with MBC. In addition, community-level interventions to improve breast cancer awareness, strengthen early detection programs, and ensure equitable access to oncology services remain important priorities, particularly in LMICs.
